# Assessing caries status according to the CAST instrument and WHO criterion in epidemiological studies

**DOI:** 10.1186/1472-6831-14-119

**Published:** 2014-09-26

**Authors:** Ana Luiza de Souza, Soraya Coelho Leal, Ewald M Bronkhorst, Jo E Frencken

**Affiliations:** Department of Global Oral Health, College of Dental Sciences, Radboud University Medical Centre, Nijmegen, The Netherlands; Department of Dentistry, School of Health Sciences, University of Brasília, Campus Universitário Darcy Ribeiro, Asa Norte, Brasília, DF CEP 70710-90 Brazil; Department of Preventive and Restorative Dentistry, College of Dental Sciences, Radboud University Medical Centre, Nijmegen, The Netherlands

**Keywords:** Caries assessment spectrum and treatment, CAST, Caries epidemiology, Dental caries/diagnosis*, Disease progression, DMF index

## Abstract

**Background:**

The Caries Assessment Spectrum and Treatment (CAST) is a new epidemiological instrument for detection and treatment of dental caries. Worldwide, the WHO criterion constitutes the epidemiological tool most commonly used for caries detection. The objective of the present study is to determine the levels of similarity and difference between the CAST instrument and WHO criterion on the basis of caries prevalence, dmf/DMF counts, examination time and reporting of results.

**Methods:**

An epidemiological survey was carried out in Brazil among 6-11-year-old schoolchildren. Time of examinations was recorded. dmft, dmfs, DMFT and DMFS counts and dental caries prevalence were obtained according to the WHO criterion and the CAST instrument, as well the correlation coefficient between the two instruments.

**Results:**

Four hundred nineteen children were examined. dmft and dmfs counts were 1.92 and 5.31 (CAST), 1.99 and 5.34 (WHO) with correlation coefficients (*r)* of 0.95 and 0.93, respectively. DMFT and DMFS counts were 0.20 and 0.33 (CAST), 0.19 and 0.30 (WHO), with *r* = 0.78 and *r* =0.72, respectively. Kappa coefficient values for intra-examiner consistency were CAST = 0.91-0.92; WHO = 0.95-0.96 and those for inter-examiner consistency were CAST = 0.90-0.96; WHO = 0.94-1.00. Mean time spent on applying CAST and WHO were 66.3 and 64.7 sec, respectively p = 0.26. The prevalence of dental caries using CAST (codes 2, 5-8) and the WHO criterion for the primary dentition were 63.0% and 65.9%, respectively, and for the permanent dentition they were 12.7% and 12.8%, respectively.

**Conclusions:**

The CAST instrument provided similar prevalence of dental caries values and dmf/DMF counts as the WHO criterion in this age group. Time spent on examining children was identical for both caries assessment methods. Presentation of results from use of the CAST instrument, in comparison to WHO criterion, allowed a more detailed reporting of stages of dental caries, which will be useful for oral health planners.

## Background

Oral health surveys are conducted to obtain information about the prevalence and extent of oral health conditions, with the aim of designing oral health policies and programmes. Various sets of criteria exist for assessing dental caries. The one recommended by the World Health Organisation (WHO) has been used most frequently. It considers dental caries to be an unmistakable cavitated lesion into dentine [1]. The advantages of the WHO criterion include ease in mastering the criterion and its use in practice, the high levels of agreement among examiners and the possibility for comparing results collected from many populations worldwide over long periods. A disadvantage is the absence of codes for recording caries lesions in enamel. Another disadvantage is the difficulty for differentiating caries lesions in dentine that can be treated restoratively from those that require more complicated treatment.

A new caries assessment instrument termed Caries Assessment Spectrum and Treatment (CAST) was promulgated in 2011 [2]. It was designed for use in international epidemiological surveys and permits registration of sound teeth, sealants, restorations, enamel and dentine caries lesions, advanced stages of caries lesions into the pulp and tooth-surrounding tissues, and teeth lost due to dental caries (Table 1) [2, 3]. The CAST instrument differs from other caries assessment instruments by the fact that the codes are ordered in increasing level of severity of the effects of the caries process. In this hierarchical order, a tooth surface containing a sealant and one having a restoration are considered healthy surfaces. CAST, like other caries assessment instruments regards a caries lesion, whether in enamel or dentine, as a more severe condition than a sealed or restored surface. CAST allows the reporting of teeth with morbidity and mortality whereby a tooth lost through the caries process is considered ‘not diseased anymore’. The implications of the rationale upon which CAST has been built leads to a different calculation of the prevalence of dental caries, which is not based on the presence of a dmf/DMF count of ≥1 but on that of d/D ≥1. CAST codes allow the calculation of a dmf/DMF count of an individual tooth, in comparison to those of other caries assessment indices [3].

**Table 1:** **The codes and descriptions of the hierarchical CAST instrument**

Characteristic	Code	Description
Sound	0	No visible evidence of a distinct carious lesion is present
Sealant	1	Pits and/or fissures are at least partially covered with a sealant material
Restoration	2	A cavity is restored with an (in)direct restorative material
Enamel	3	Distinct visual change in enamel only. A clear caries related discolouration is visible, with or without localised enamel breakdown
Dentine	4	Internal caries-related discolouration in dentine. The discoloured dentine is visible through enamel which may or may not exhibit a visible localised breakdown of enamel
	5	Distinct cavitation into dentine. The pulp chamber is intact
Pulp	6	Involvement of the pulp chamber. Distinct cavitation reaching the pulp chamber or only root fragments are present
Abscess/Fistula	7	A pus containing swelling or a pus releasing sinus tract related to a tooth with pulpal involvement
Lost	8	The tooth has been removed because of dental caries
Other	9	Does not correspond to any of the other descriptions

CAST was validated for face, content and construct, and its reproducibility was tested clinically among three age groups. Face and content validity were determined using the RAND modified e-Delphi consensus method, by a group of 56 experienced epidemiologists from 24 countries [4]. Construct validity was determined by comparing CAST codes obtained from visual examination with those obtained through examining histology and micro-computed tomography images as gold standards according to the Downer caries assessment criterion [5]. The results of the reproducibility tests of CAST among primary and permanent teeth in children and adults showed that training and calibration exercises were performed well and that the agreement in caries conditions scoring, using CAST, among examiners was high [6]. Therefore, the CAST instrument appears to be a promising tool for use internationally in oral health surveys. It is currently being used in populations of different age groups and backgrounds in a number of countries exemplified by the study from Poland [7].

In most countries, the results of studies investigating caries experience are predominantly expressed in mean dmf/DMF scores. Many of such studies have applied the WHO criterion, which can therefore be considered a reference. Although it appears that a mean dmf/DMF score can be retrieved from the data collected through use of CAST, this assumption has not been investigated. In order to further understand the characteristics of CAST for use in population groups, it is important to determine the time needed to perform an examination using the CAST instrument in comparison to that of the WHO criterion. Such a study has also not previously been performed. As CAST and WHO caries detection criteria differ significantly in their descriptions, reporting of results is bound to be presented in different ways. As the results of studies that have used CAST have not been presented yet, an attempt is made to present these in comparison with those obtained according to the WHO criterion.

This study aimed to determine the levels of similarity and difference between the CAST instrument and WHO criterion on the basis of caries prevalence, dmf/DMF counts, performance time and result reporting.

## Methods

A 4-year mixed-longitudinal cohort study, initially covering 6-7-year-old children in Paranoá, a district of Brasília, Brazil, started in 2009 in six public primary schools [8]. In 2011 and 2013, these children were re-examined, together with two new cohorts of 6-7-year-olds. In 2013, one of the schools was selected for the current comparison study and all children from the three age cohorts were examined according to both the CAST instrument and the WHO criterion.

The current study was approved by the Ethics Committee of the University of Brasília (CEP-FM 014/2011). Parents or legal guardians were given a consent form explaining the nature of the study. Only those children who returned the duly signed form and agreed to be examined were included.

### Examiner training

A senior epidemiologist (JEF) conducted training and calibration sessions. Training comprised a theoretical explanation about the CAST instrument (1.5 hours) and a practical session (2 hours) in which a total of 20 extracted teeth were examined and scored by each of the three examiners. Individual scores were compared and, in case of a difference, examiners discussed the scores until consensus was reached. This process was repeated to achieve good agreement amongst examiners. In order to be trained in the use of the WHO criterion, examiners read and discussed the WHO manual [1] before starting the practical session. The examiners each examined 20 children and discussed their findings, to resolve any misunderstanding amongst them in scoring caries lesions.

Having been trained, examiners were calibrated for the CAST instrument and the WHO criterion, the Visible Plaque Index [9] and Gingival Bleeding Index [10] during an 8-hour session. They examined 14 children of the same age as those included in the main study, drawn from the same socio-economical background. The kappa-coefficient values for inter-examiner agreement at the end of the calibration session ranged from 0.80 to 0.96 (CAST) and from 0.94 to 0.96 (WHO), while the percentage of agreement among the examiners ranged from 93.9% to 97.2% and from 96.1% to 98.4% for the CAST instrument and the WHO criterion, respectively. These findings were considered sufficient for the comparison study to start.

### Oral examination

The examinations were conducted on school premises by three trained and calibrated examiners assisted by three trained recorders, using portable dental equipment and artificial light. Two examiners had experience in conducting epidemiological surveys. The senior epidemiologist was present during the first examination week, to provide assistance to the examiners in case of doubts and for discussing cases.

The examination started with assessment of the presence of toothache, plaque and gingival bleeding. Thereafter, patients’ teeth were cleaned by the examiners using a toothbrush, dental floss and/or gauzes. Dental caries status was then assessed using a mirror handle with a battery-powered built-in light source (MirrorLite®, Kudos, Hong Kong) and a CPI probe. As recommended for application of CAST, the tooth surface was not air-dried [2, 3] but, when necessary, excess saliva was removed with cotton rolls or gauzes. Examination time was recorded by the assistants using a chronometer from the moment the examiner picked up the instruments and called out ‘start’ , until the examiner concluded the examination and called out ‘finished’.

The CAST instrument was used in the first examination. The examination in which the WHO criterion was used was performed one to three weeks later.

### Statistical analysis

Data analysis was performed by an oral statistician using the software IBM SPSS for Windows, version 21.0 (Chicago, IL, USA). Table 2 presents the codes and descriptions that were used for calculating mean dmf/DMF scores for comparison between the CAST instrument and the WHO criterion. The respective dmf/DMF counts obtained through using the WHO criterion were calculated as follows: decayed = code B or 1 and code C or 2, missing = code E or 4 and filled = code D or 3. The dmf/DMF counts obtained through use of the CAST instrument, for comparison with those obtained through using the WHO criterion, were calculated as follows: decayed = code 5-7, missing = code 8 and filled = code 2. In the present study, the prevalence of dental caries according to CAST is presented in two ways: 1) according to the CAST rationale by considering only current caries lesions (either codes 4-7 for dentine lesions or codes 3-7 for enamel and dentine lesions) and; 2) for comparison with the WHO criterion by considering caries experience (codes 2, 5-8).

**Table 2:** **CAST and WHO codes and descriptions**

CAST		WHO		
Primary and permanent teeth		Primary teeth	Permanent teeth	Description
Code	Short description	Code		
0	Sound	A	0	Sound
1	Sealant, partial or total	F	6	Fissure sealant
2	Restoration, direct or indirect	D	3	Filled, no decay
		G	7	Bridge abutment, special crown or veneer implant
3	Enamel lesion	A	0	Sound
4	Dentine lesion	A	0	Sound
5	Cavitated dentine lesion	B	1	Decayed
		C	2	Filled, with decay
6	Pulpal involvement	B	1	Decayed
7	Abscess/Fistula	B	1	Decayed
8	Missing due to caries	E	4	Missing, as a result of caries
9	Other	-	9	Not recorded
		-	5	Missing, any other reason
		-	8	Unerupted tooth (crown)
		T	T	Trauma

Agreement among examiners in mean dmft, dmfs, DMFT and DMFS scores obtained from application of the CAST instrument and the WHO criterion was calculated using unweighted kappa statistics (κ), standard error (SE) and percentage of agreement (P_o_), and analysed using paired sample test and Pearson correlation coefficient (*r*). A statistically significant difference was set at p ≤ 0.05.

## Results

### Background information

In 2013, the survey examined a total of 2416 children. Of these, 419, aged 6-11-years, were examined. Both the CAST and WHO criterion were used. A total of 64 and 62 children were re-examined using the CAST instrument and the WHO criterion, respectively one to three weeks after the first examination was performed. The mean CAST instrument and WHO criterion time and standard deviations in examining the children, were 66.3 ± 32.1 sec and 64.7 ± 31.0 sec, respectively (p = 0.26).

### Reproducibility of data

Table 3 shows kappa-coefficient values, standard error and percentage of agreement of intra- and inter-examiner consistency tests in assessing primary and permanent dentitions at surface level according to the CAST instrument and WHO criterion. For CAST, the kappa coefficient values for the intra-examiner agreement ranged from 0.90 to 0.96 and those for the inter-examiner agreement were 0.91 and 0.92. For the WHO criterion, the kappa coefficient values for intra-examiner agreement ranged from 0.94 to 1.00 and those for inter-examiner agreement were 0.95 and 0.96. The percentage of agreement amongst examiners for application of the CAST instrument was ≥ 94.8% and for application of the WHO criterion it was ≥ 97.4%.

**Table 3:** **Intra- and inter-examiner consistency of assessment of primary and permanent dentitions at surface level using the CAST instrument and WHO criterion**

		CAST				WHO			
Intra-examiner		n	κ	SE	P_o_	n	κ	SE	P_o_
	Examiner 1	6912	0.94	0.004	97.4	723	1.00	0.00	100
	Examiner 2	8851	0.90	0.004	94.8	382	0.98	0.01	99.4
	Examiner 3	8208	0.96	0.003	96.6	538	0.94	0.01	97.4
Inter-examiner									
	Examiner 1-2	8424	0.92	0.004	97.0	4838	0.96	0.01	98.3
	Examiner 2-3	7992	0.91	0.004	95.3	4848	0.95	0.01	97.9
	Examiner 1-3	6264	0.92	0.005	95.6	5204	0.95	0.01	97.8

n = number of surfaces; κ = kappa-coefficient value; SE = standard error; P_o_ = percentage of agreement.

### Similarity in study findings between the CAST instrument and WHO criterion

Table 4 shows mean dmft, dmfs, DMFT and DMFS scores obtained from use of the CAST instrument and WHO criterion, with corresponding 95% confidence interval (CI) of the difference and correlation coefficient. There were no statistically significant differences in mean dmft, dmfs, DMFT and DMFS scores obtained from using both caries assessment criteria. The correlation coefficients were all high. The prevalence of dental caries among the sampled children, representing dmf/DMF counts and calculated according to the CAST instrument codes 2, 5-8 and the WHO criterion for the primary dentition, was 63.0% and 65.9%, respectively, and for the permanent dentition it was 12.7% and 12.8%, respectively, showing a high level of agreement.

**Table 4:** **Mean dmft, dmfs, DMFT and DMFS scores obtained from using the CAST instrument (codes 2, 5-8) and the WHO criterion with corresponding 95% Confidence Interval (CI) of the difference and correlation coefficient (** ***r*** **)**

	CAST	WHO			
			95% CI of the difference		***r***
dmft	1.92	1.99	-0.14	0.01	0.95
dmfs	5.31	5.34	-0.29	0.23	0.93
DMFT	0.20	0.19	-0.03	0.05	0.78
DMFS	0.33	0.30	-0.05	0.11	0.72

### Differences in study findings between CAST instrument and WHO criterion

**Figure 1:**
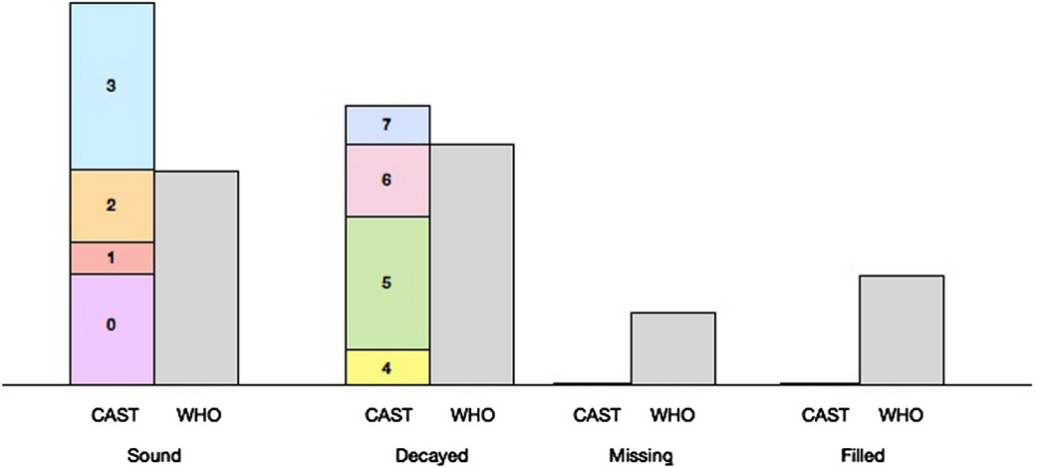
**Conceptual difference in calculating the prevalence of dental caries according to CAST (C) instrument and WHO (W) criterion.**

The conceptual difference in determining the prevalence of dental caries according to the CAST instrument and the WHO criterion is presented in Figure 1. Following the rationale of CAST, calculating the prevalence of dental caries is confined to the presence of caries lesions only; either to lesions including the dentine (codes 4-7) or to those including both enamel and dentine (codes 3-7).

The prevalence of dental caries (dentine lesions) in primary dentition of the sampled children, calculated according to the CAST instrument and the WHO criterion, was 57.9% and 65.9%, respectively and for the permanent dentition it was 8.7% and 12.8%, respectively. The proportion of dentine caries lesions assessed as being restorable (codes 4,5) was 81.2% for primary teeth and 89.7% for permanent teeth. Prevalence of dental caries including enamel and dentine lesions according to CAST, for the primary and permanent dentition was 72.5% and 35.7%, respectively. The WHO criterion cannot be used for such a calculation.

Table 5 shows the hierarchical order of CAST codes before and after use of conventional dental treatments. It implies that, after all caries treatment has been provided to the children, the prevalence of dental caries in primary and permanent dentitions would return to zero if CAST were used to report data. If the WHO criterion was used to assess the caries situation after treatment, the prevalence of dental caries would continue to be 65.9% and 12.8% for primary and permanent dentitions, respectively.

**Table 5:** **The hierarchical order of CAST codes before and after use of conventional treatments**

Code		Status	Treatment	Status after Tx	Code at end of Tx
0		Healthy	Maintenance care	Healthy	0
1	S	Healthy	Maintenance care	Healthy	1
2	R	Healthy	Maintenance care	Healthy	2
3	E	Premorbidity	Maintenance/Preventive care	Premorbidity/Healthy	3 or 1
4	D	Morbidity	Sealant/Restorative care	Healthy	1 or 2
5	D	Morbidity	Restorative care	Healthy	2
6	P	Morbidity	Pulp treatment or extraction	Healthy	2 or 8
7	P	Morbidity	Pulp treatment or extraction	Healthy	2 or 8
8	L	Mortality	Nothing? Rehabilitative care?	Not diseased	8

Tx = treatment.

### Reporting caries status according to CAST

Table 6 shows the frequency distribution of the CAST codes for the primary and permanent dentitions of 6- to 11-year-olds.

**Table 6:** **Frequency distribution (%) of 6- to 11-year-olds having teeth scored by CAST codes for the primary and permanent dentitions**

	Primary		Permanent	
Frequency	≥ 1	≥ 3	≥ 1	≥ 3
CAST code				
0	92.4	87.2	94.7	84.5
1	0.0	0.0	10.3	6.7
2	13.8	1.7	4.3	0.7
3	50.6	15.8	30.3	12.7
4	5.5	0.5	0.0	0.0
5	50.6	19.4	7.6	0.2
6	18.9	4.4	1.2	0.0
7	3.8	0.0	0.0	0.0
8	6.0	0.5	0.0	0.0

In the primary dentition more than half of the children (50.6%) had at least one tooth with a caries lesion in the enamel and 15.8% of them had at least three teeth with such a condition. The percentage of children with at least one tooth with a caries cavity, confined to the dentine (code 4,5), was 56.1% and the percentage with at least one tooth with a caries cavity including the pulp (code 6) 18.9%. At least three teeth with caries cavities confined to the dentine were observed in 19.9% of the children, whereas 4.4% of the children had three or more teeth with a caries cavity that had reached the pulp. At least one abscessed primary tooth was observed in 3.8% of the children examined.

In the permanent dentition, at least one sealant-containing tooth was observed in 10.3% of the children and one restored tooth in 4.3%. At least one tooth with a caries lesion in enamel (code 3) was observed in 30.3% of the children examined, and one tooth with a caries cavity confined to the dentine (code 4,5) in 7.6% of them.

## Discussion

Based on the mean dmf/DMF scores and on the prevalence of dental caries, the present study did not show a significant difference between the CAST instrument and the WHO criterion results. This implies that for the age group of 6-11 years, the caries prevalence and caries experience obtained through use of the CAST instrument can be compared with those obtained through using the WHO criterion. A very recently published caries epidemiological survey on occlusal surfaces of permanent first molars of 6-8-year-old children appear to confirm this finding [11]. Whether the high level of similarity between CAST and WHO criterion is also present in other age groups and in populations with different treatment patterns is unknown and needs to be investigated. For example, in populations with a high prevalence of restored teeth that also contain enamel caries lesions, a single tooth is categorised as restored according to the WHO criterion, while such a tooth is categorised as an enamel caries lesion when CAST is used. This difference is due to the hierarchical order within CAST that considers a tooth containing an enamel caries lesion as in a more severe condition than a restored tooth. This novelty in CAST might affect the level of agreement in dmf/DMF counts between the two caries assessment instruments and is dependent upon the frequency of occurrence of combinations of caries codes in a tooth.

The time taken to perform the examinations did not differ between the two caries assessment instruments. As the time needed in using CAST to examine children in other investigations has not yet been reported, comparing the result of the present study with those of others is not possible. Information about the examination time needed for using the WHO criterion appears to be scarce. Using the WHO criterion in examining 3-5-year-olds has taken on average 1.90 minutes [12], which is longer than the time recorded in the present study (66 sec) and could be due to the older age of children in the present study, who might have been more cooperative than younger ones.

The prevalence of a disease or condition is determined by the number of individuals in a population affected by that disease or condition. Prevalence may vary, depending on the cause of the disease/condition and the effect of treatments rendered. However, the prevalence of dental caries currently is determined in a different way. Not only is the prevalence based on individuals affected by the disease, it is also based on individuals who had been affected and received treatment. This is because the calculation of the prevalence of dental caries is based on the dmf/DMF counts of individuals who constitute the population under study. Therefore, merely using the dmf/DMF count in calculating the prevalence of dental caries provides an erroneous picture of the actual situation of the disease in an individual. Individuals who have had a stable dentition with three restorations and some enamel caries lesions that never progressed into cavitation over a period of forty years are not considered healthy, according to the calculations currently in use for determining the prevalence of dental caries. The dental profession has been using this approach, most probably since the introduction of the dmf/DMF index by Klein and Palmer [13] and is still teaching it. Even the innovators of the ICDAS system decided to use the dmf/DMF index in reporting their results [8, 14–16]. It goes without saying that the traditional way of calculating the prevalence of dental caries does not depict well the efforts made by the dental community in serving the population, as a lower prevalence cannot be shown post treatment. This way of calculating the prevalence of dental caries is unwarranted and ought to be rectified. In an attempt to achieve greater accuracy, CAST was developed. In this assessment instrument, only teeth that have a cavitated dentine caries lesion and those that show its consequences (CAST codes 5-7) are considered diseased and included in the calculation of the prevalence of dental caries. A restored tooth and an extracted tooth are not included, because the first one has been treated and the second one is not considered diseased anymore. Following this rationale, a population that, e.g. had a prevalence of dental caries of 45 percent before undergoing treatment, will reach a prevalence of below 45 percent after treatment, showing the effect of the treatments rendered.

The cut-off point for determining the level of dental caries, whether in enamel or in dentine, can also be made when CAST is used. Enamel caries lesions in the CAST instrument are represented by one category only, in contrast to the one used in the ICDAS system, which uses three different stages. When the latter is used, the prevalence of dental caries is unnecessarily inflated if the lowest caries lesion code is used as a cut-off point for determining the prevalence of dental caries [8, 14, 15]. This should be avoided. Marthaler [17], after experiencing difficulties in using a two-coded caries lesion system in enamel, reduced it to one.

Thus, reporting of the caries status according to CAST allows for the presentation of a pre-morbidity stage that calls for preventive actions. Furthermore, CAST also distinguishes dentine caries lesions that can be restored from those that are beyond treatment with a restoration alone. These caries conditions are not included in the WHO criterion, which is a disadvantage. For example, the last epidemiological survey conducted in Brazil, which used the WHO criterion, concluded that about 80% of decayed primary teeth in 5-year-olds remained untreated [18]. Having these results as a reference, health planners are unable to provide a realistic overview of the kind of treatments needed and consequently, cannot accurately calculate the amount of (restorative) dental materials, instruments, equipment and budget required to improve the situation adequately.

The present study reported a low proportion of children having teeth affected with a caries lesion reaching the pulp, which in most cases would require an extraction. It further showed a high prevalence of children with teeth having an enamel caries lesion requiring preventive measures, dental health education and regular surveillance. Additionally, a substantial number of children required a restoration or an ultraconservative treatment [19], showing that the current oral health system is not capable of providing curative care for the schoolchildren in Paranoá. Therefore, using CAST allows health authorities to have an available tool that enables them to plan oral health care programmes better than the WHO criterion makes possible.

## Conclusions

DMFT, DMFS, dmft and dmfs counts and dental caries prevalence calculated through use of the CAST instrument in this age group were not significantly different from those obtained through use of the WHO criterion. The time spent on examining the mixed dentition of these children when using both caries assessment methods was identical. The CAST instrument provides a realistic reproduction of the prevalence of dental caries in populations and it facilitates development of an adequate health policy and dental care planning for the population.
